# Conserved gut microbiomes with cross-species spillover between sympatric Neotropical stingless bees and honey bees

**DOI:** 10.1128/aem.02483-25

**Published:** 2026-04-17

**Authors:** Karen Luisa Haag, Luiza Quadro Stein, Carlos Gustavo Nunes da Silva, Florent Mazel, Aiswarya Prasad, Philipp Engel

**Affiliations:** 1 Department of Genetics, Institute of Biosciences, Federal University of Rio Grande do Sul 124596, Porto Alegre, Brazil; 2 Department of Morphology, Institute of Biological Sciences, Federal University of Amazonas 67892, Manaus, Brazil; 3 Department of Fundamental Microbiology, University of Lausanne 54172, Lausanne, Switzerland; Norwegian University of Life Sciences, Ås, Norway

**Keywords:** stingless bee, microbiome, spillover, host specificity

## Abstract

Stingless bees (Meliponini) are ecologically vital pollinators with deep cultural and economic importance in the Neotropics; however, the biogeographic structure of their gut microbiota and the extent of microbial exchange with managed honey bees (*Apis mellifera*) remain insufficiently understood. Using full-length 16S rRNA gene sequencing of individually sampled workers from 167 colonies across Brazil, we compared gut bacterial communities of *Melipona quadrifasciata* and *Melipona mondury* with those of co-occurring *A. mellifera* through an integrated taxonomic, phylogenetic, and community ecological framework. The core microbiota of *Melipona* species was dominated by *Lactobacillus*, *Bifidobacterium*, *Apilactobacillus*, *Bombella*, and *Floricoccus*, whose relative abundances covaried inversely with a set of low-prevalence taxa. Although the core communities of stingless bees overlapped only partially with those of honey bees, both groups displayed comparable alpha- and beta-diversity dispersion, suggesting broadly similar assembly dynamics. Notably, 6% of all amplicon sequence variants (ASVs) were shared across hosts, encompassing nearly all canonical honey bee symbionts, consistent with frequent cross-species spillover. Among these, several *Snodgrassella* ASVs—typically rare in these stingless bee species—reached high abundance in *M. quadrifasciata* and formed a deeply divergent clade (~96% 16S rRNA identity to *Snodgrassella alvi*). These patterns indicate that human-mediated management practices, such as mixed apiaries and artificial feeding, create ecological opportunities for interspecific microbial exchange. Overall, our results show that stingless bee gut microbiomes are compositionally stable yet ecologically permeable, shaped jointly by long-term host specificity and recent anthropogenic contact.

**IMPORTANCE:**

Stingless bees are key pollinators in tropical ecosystems and hold long-standing cultural significance in the Neotropics; however, their microbiomes remain far less studied than those of managed honey bees. Understanding how gut bacterial communities vary across landscapes, and whether microbes move between native and non-native hosts, is essential for predicting the ecological consequences of increasing meliponiculture and urban beekeeping. Our study reveals that stingless bee gut microbiota are generally stable and host-associated but nonetheless acquire bacterial symbionts typical of honey bees, indicating that human management practices facilitate cross-species microbial transmission. These findings broaden current knowledge of bee–microbe evolution by showing that gut symbiont boundaries are not fixed but can become permeable under anthropogenic influence. This has important implications for pollinator health, conservation, and biosecurity as managed and native bees increasingly co-occur in human-modified environments.

## INTRODUCTION

The gut microbiota of social bees, such as bumble bees, honey bees, and stingless bees, is taxonomically simple and becomes established soon after adult emergence (1, 2). These symbionts contribute to the digestion of pollen- and plant-derived polysaccharides (3, 4), detoxification of secondary plant metabolites (5), host learning and memory (6, 7), and protection against pathogens (8–10). Because their microbiota are dominated by a small number of host-specific lineages, social bees have become valuable models for studying the evolution, maintenance, and host interactions of animal-associated microbial communities (11). However, our understanding of these symbioses is shaped overwhelmingly by the Western honey bee (*Apis mellifera*), the most widely studied and heavily managed bee species. The honeybee gut microbiota is remarkably consistent: five to nine dominant genera, including *Gilliamella, Snodgrassella, Bombilactobacillus, Lactobacillus, Bifidobacterium, Frischella, Bombella, Bartonella,* and *Commensalibacter*, account for most bacterial cells in the gut of an adult worker bee (12). Most of these bacteria have been cultured, and studies with gnotobiotic honeybees have started revealing their relative contribution to interactions with the host (7, 13, 14). Different honey bee species harbor gut communities that are similar in composition but host-specific, that is, they share several bacterial genera, but these are mostly represented by distinct species and strains (15, 16). Some of the same bacterial genera are also present in the gut microbiota of bumble bees and stingless bees, pointing to a shared evolutionary history. However, much less is known about the gut microbiota of these groups of social bees. In particular, knowledge about stingless bees (Meliponini), a taxonomically diverse and ecologically critical clade of tropical pollinators, remains limited. Only recently have studies begun to describe their gut microbiota composition, and most reports are geographically limited (15, 17–20). We still know little about how variable stingless bee gut communities are across the hosts’ broad geographic ranges, whether they display the same strong host specificity seen in *A. mellifera*, and which ecological or anthropogenic factors might drive community turnover.

Humans have interacted with social bees for millennia. The collection of honey by humans dates back to the Neolithic (21, 22), and stingless bees were managed by pre-Columbian civilizations long before the worldwide spread of *A. mellifera* by European settlers (23). In the Neotropics, meliponiculture and apiculture frequently occur side by side, despite *A. mellifera* being introduced to the region and *Melipona* species being native and geographically restricted to the Americas. Managed colonies of honey bees and stingless bees are frequently kept in close proximity, transported across regions, and manipulated for honey production. Artificial feeding, including practices such as sugar syrup supplementation and, in some cases, the use of *A. mellifera* honey, may occur in stingless bee management during periods of resource scarcity. Such interventions can create ecological contact zones where gut microbes from one host may encounter and potentially colonize another. Evidence for microbiota spillover in bees remains limited but is growing, and recent studies in honey bees suggest that some bacterial taxa may be shared among closely related hosts (16). Some floral- and environment-associated bacteria appear in multiple bee hosts and colony environments (17, 19, 24), and there are scattered reports of *Snodgrassella* or *Lactobacillus* strains occurring outside their typical hosts (18, 25, 26). However, to the best of our knowledge, no broad-scale, strain-level test has addressed whether gut symbionts from managed honey bees infiltrate native stingless bees or vice versa.

High-throughput sequencing of full-length 16S rRNA genes using PacBio now enables the detection of bacterial community composition at the subspecies or strain level, allowing researchers to characterize fine-scale diversity patterns within and across host species. Here, we implement these advances to investigate the gut microbiota of two widespread stingless bees, *Melipona quadrifasciata* and *Melipona mondury*, sampled alongside sympatric *A. mellifera* colonies across Brazil. Our primary goals were (i) to quantify within- and between-host variation in the gut microbiota of these two important stingless bee species across a broad geographic range, (ii) to test whether these communities are more environmentally assembled or variable than those of honey bees, and (iii) to assess the extent and direction of cross-species bacterial sharing and potential spillover, particularly in human-managed settings where honey bees and stingless bees coexist in close proximity.

## RESULTS

### Full-length 16S rRNA gene amplicon sequencing of the gut microbiota of sympatric *A. mellifera*, *M. quadrifasciata*, and *M. mondury*

**Fig 1:**
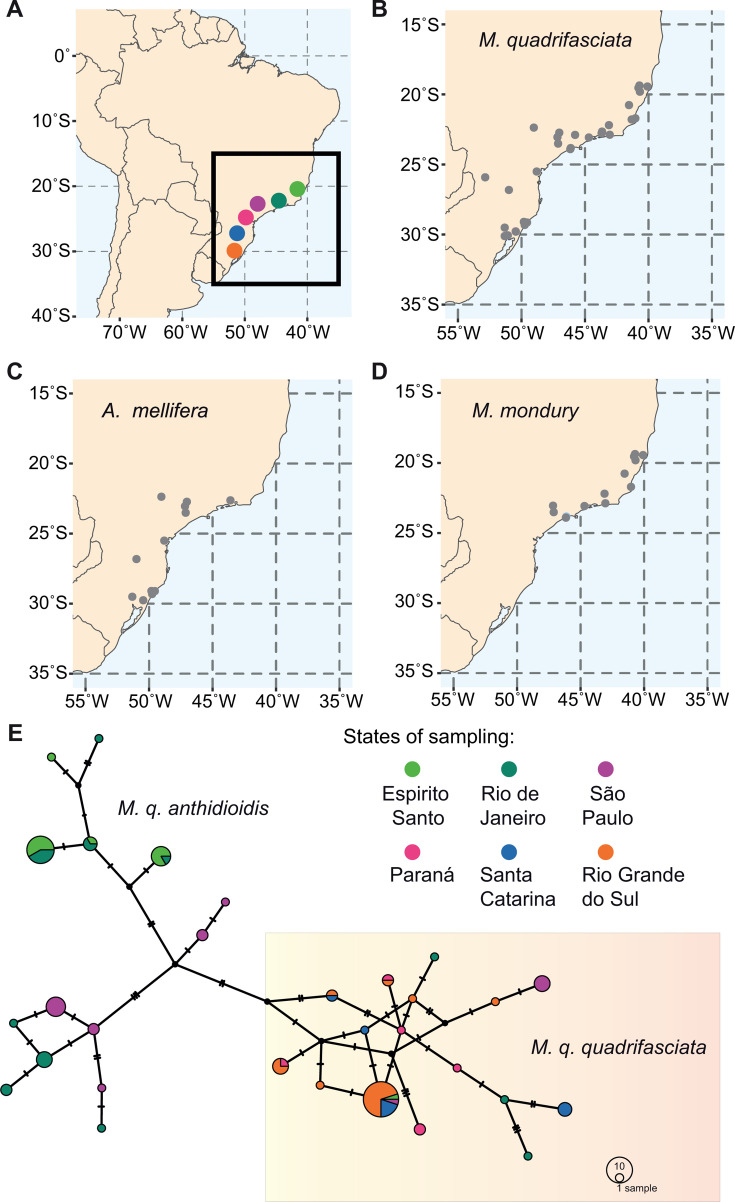
Origin of bees analyzed in our study. (**A**) Map showing the region of Brazil where field excursions were made (squared area). Colored circles and their respective two-letter codes refer to the Brazilian states where sampling sites are located. (**B–D**) Distribution of sampling sites for each bee species within the region shown in panel A. (**E**) Haplotype network used to identify *M. quadrifasciata* subspecies (circle sizes indicate abundance and colors indicate geographic origin).

We sampled 167 managed colonies of three eusocial bee species (41 *A. mellifera*, 37 *M. mondury,* and 89 *M. quadrifasciata*) from a total of 30 locations, covering most of the known geographic range of *M. quadrifasciata* (Fig. 1). For three of the 30 locations, all three species were sampled, while in 24 locations, two of the three species were sampled. Of the 89 colonies of *M. quadrifasciata*, 46 and 43 were identified as subspecies *M. q. quadrifasciata* and *M. q. anthidioidis*, respectively. From each colony, one adult worker bee was used for gut microbiota analysis. To obtain high taxonomic resolution beyond the bacterial species level, we used PacBio full-length 16S rRNA gene amplicon sequencing. We obtained, on average, 14,628 (min = 7,328, max = 36,752) reads per sample and detected a total of 4,376 ASVs with an average of 59 (min = 13, max = 230) ASVs per sample. A total of 658 ASVs (15%) could not be annotated at the genus level. The largest proportion of unassigned reads at the genus level in our data set comes from *M. quadrifasciata* (78.4%), followed by *M. mondury* (19.8%) and *A. mellifera* (1.9%). Most of the unassigned reads belong to the Lactobacillaceae (55.5%, corresponding to 289 ASVs) and Acetobacteraceae (31%, 81 ASVs).

### Host identity and location explain gut microbiota composition across the three sampled bee species

**Fig 2:**
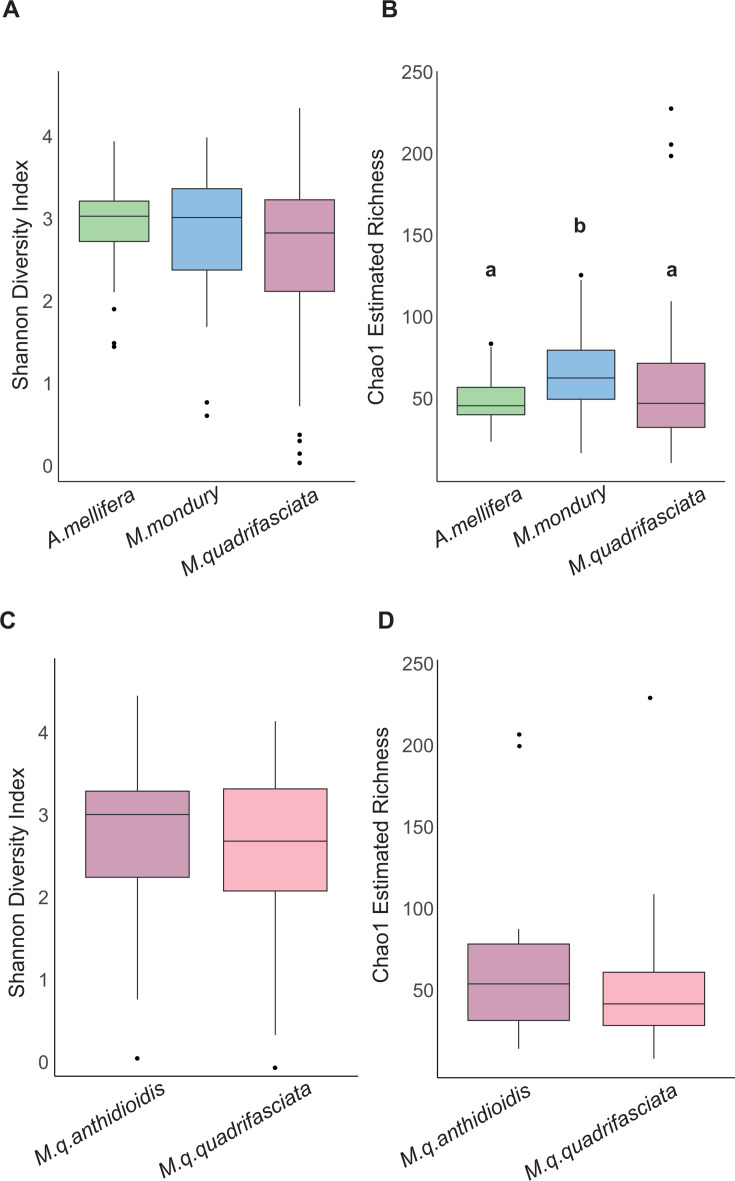
Alpha diversity estimates. Distribution of Shannon diversity index and Chao1 richness estimator obtained for bacterial communities associated with each bee species (**A and B**) as well as the subspecies of *M. quadrifasciata* (**C and D**). Different letters indicate significant differences based on Kruskal–Wallis tests, followed by Dunn tests, *P* < 0.05.

To assess differences in overall community diversity between the three bee species, we first looked at alpha-diversity. Shannon diversity index (SDI), which accounts for evenness, allowing the detection of community shifts driven by changes in relative dominance, was not significantly different between the three bee species (Fig. 2A). However, the Chao1-estimated richness (CER), which emphasizes the richness of rare ASVs, was slightly higher in *M. mondury* as compared to the other two bee species (Kruskal-Wallis X^2^ = 11.076; *P* = 0.0039; Fig. 2B). CER also showed a weak correlation with latitude (Spearman’s rho = 0.1524; *P* = 0.0535), whereas SDI did not (Spearman’s rho = 0.0252; *P* = 0.7504). No difference in alpha-diversity was observed for the gut microbiota of the two subspecies of *M. quadrifasciata* (SDI, Fig. 2C, and Chao, Fig. 2D, both *P* > 0.05).

**Fig 3:**
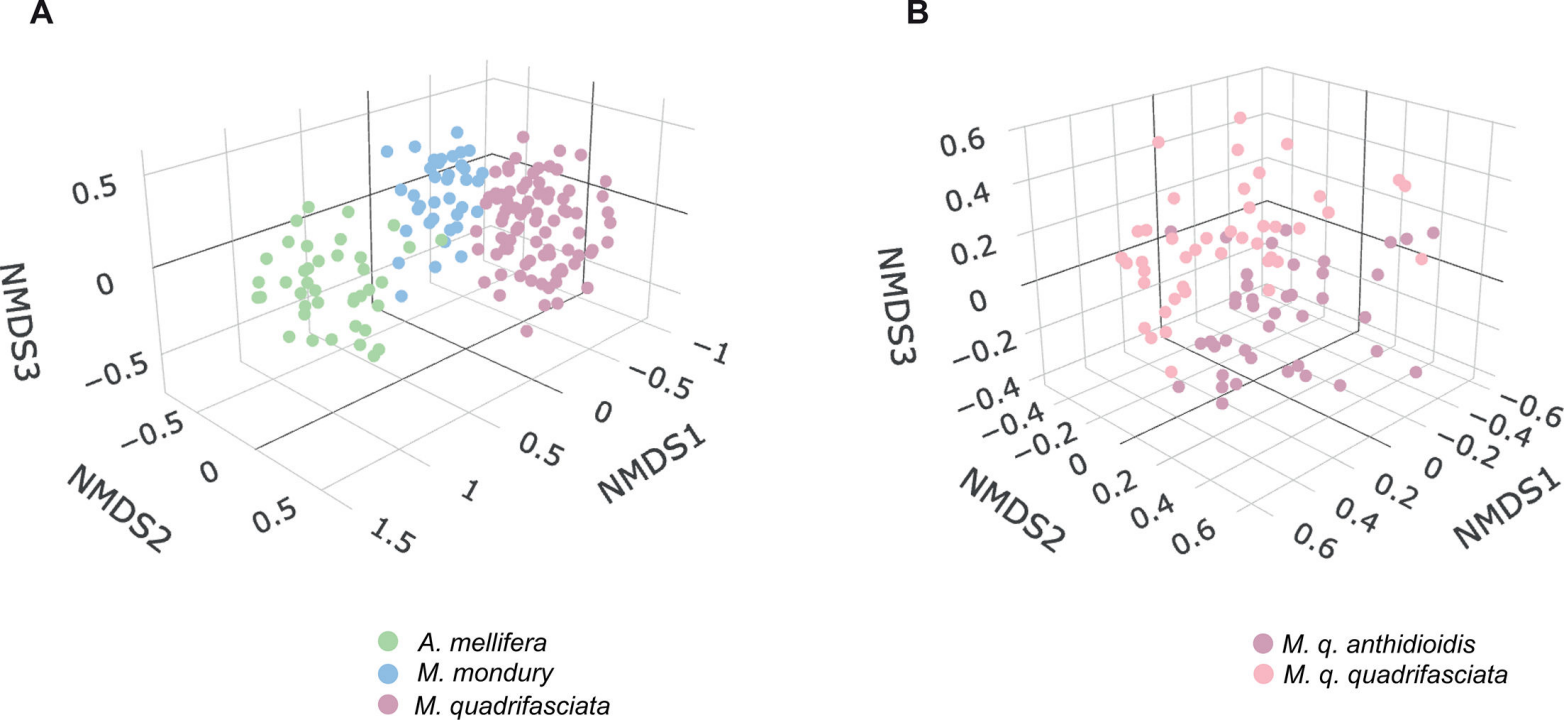
NMDS. Three-dimensional non-metric multidimensional scaling analyses of gut bacterial communities. (**A**) Analysis ran on the whole data set, with samples colored by host species (stress = 0.1099). (**B**) Analysis ran on *M. quadrifasciata* samples colored by subspecies (stress = 0.1818).

We next examined the patterns of beta-diversity across hosts and habitats. Samples from the three bee species clustered separately in a three-dimensional NMDS ordination of pairwise Bray–Curtis distances (stress = 0.1099; Fig. 3A). To formally assess the relative contributions of host and environmental factors, we used a two-factor PERMANOVA model including host identity (species or subspecies) and habitat (municipality), as well as their interaction. Importantly, each analysis was restricted to municipalities where all taxa under comparison were sampled, ensuring that observed effects were not confounded by non-overlapping geographic distributions. Across the full data set, host identity explained approximately 14% of the total variance in community composition (ω² = 0.1444; *P* = 0.001; Table 1). This pattern of host specificity remained significant even when either *A. mellifera* or *M. mondury* was excluded, with host identity still explaining ~9% of the variance in both reduced data sets (ω² = 0.0953 and ω² = 0.0856, respectively; *P* = 0.001).

**TABLE 1:** PERMANOVA of Bray–Curtis distances of gut bacterial communities performed on the whole data set as well as on different data subsets^*a*^

Data set		DF	SQ	F	w^2^	*P* value
All spp.	Host species	2	2.001	2.941	0.1444	0.001
	Habitat	3	1.853	1.816	0.0962	0.001
	Interaction	3	1.748	1.713	0.0851	0.001
*M. quadrifasciata* and *A. mellifera*	Host species	1	3.328	8.954	0.0856	0.001
	Habitat	14	7.725	1.485	0.0739	0.001
	Interaction	14	7.638	1.468	0.0716	0.001
*M. mondury* and *A. mellifera*	Host species	1	0.932	2.685	0.1010	0.001
	Habitat	3	1.828	1.755	0.1312	0.001
	Interaction	1	0.6311	1.818	0.0517	0.001
*Melipona* spp.	Host species	1	2.867	8.482	0.0953	0.001
	Habitat	13	7.793	1.774	0.1241	0.001
	Interaction	11	5.740	1.544	0.0777	0.001
*M. quadrifasciata*	Haplotype	1	1.194	3.410	0.0279	0.001
	Habitat	28	15.880	1.620	0.1712	0.001

^*a*^ The degrees of freedom (DF), sum of squares (SQ), corrected percentage of variation explained by each factor (ω^2^), and the statistical significance (*P*) are indicated.

Habitat (municipality) also exerted a significant influence on beta-diversity, explaining ~9.6% of the variance across all species (ω² = 0.0962; *P* = 0.001). The relative contribution of habitat increased when comparisons were restricted to more closely related hosts, reaching ~12.4% when only *Melipona* species were considered (ω² = 0.1241; *P* = 0.001). Importantly, the interaction term (host × habitat) was also significant in all analyses (*P*=0.001; Table 1), highlighting that the influence of local conditions differs among host taxa. Despite these patterns, there was no evidence that stingless bees’ microbiota are more environmentally structured than those of honey bees: beta-dispersion analyses revealed no significant difference in the variability of Bray–Curtis distances among hosts (ANOVA; *F* = 0.7987; *P* = 0.4517). Finally, the gut microbiotas of the two *M. quadrifasciata* subspecies (*M. q. quadrifasciata* and *M. q. anthidioidis*) were distinguishable in NMDS space (stress = 0.1818; Fig. 3B), with haplotype explaining ~3% of total variance (ω² = 0.0279; *P* = 0.001).

### Different genera dominate the gut microbiota of *M. quadrifasciata* and *M. mondury*, as compared to *A. mellifera*

**Fig 4:**
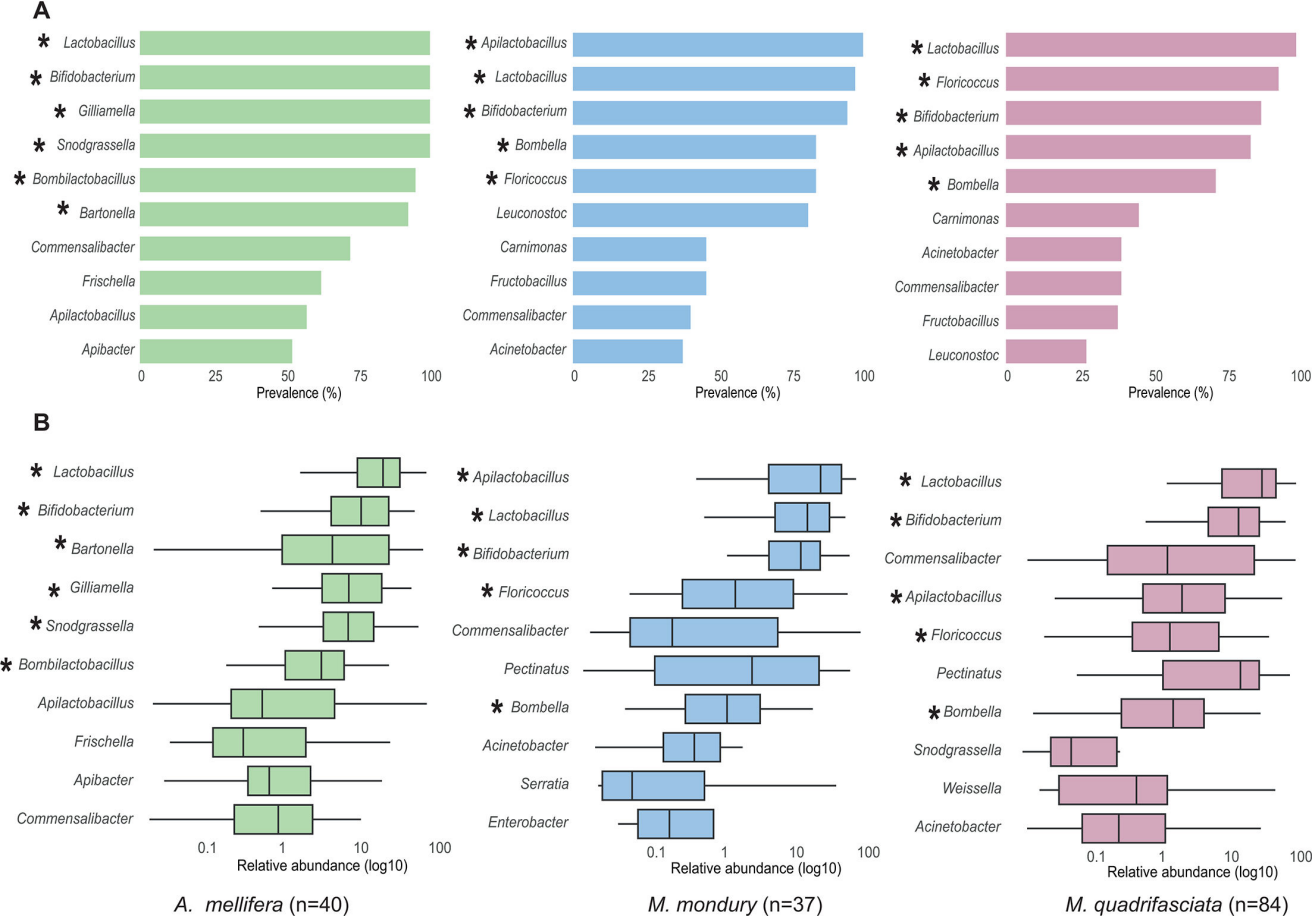
The core genera of the gut microbiota across the three bee species. The 10 bacterial genera with the highest (**A**) prevalence and (**B**) relative abundance estimates are shown in descending order of their mean values. Asterisks indicate the core microbiota members of each bee species according to our criteria (see text for details).

We next identified the core microbiota of each bee species, defining core members as genera present in >70% of individuals and with an average relative abundance of >5% (Fig. 4). For the gut microbiota of *A. mellifera,* we identified six core genera given our thresholds: *Lactobacillus*, *Bifidobacterium*, *Gilliamella*, *Snodgrassella*, *Bombilactobacillus*, and *Bartonella*. For the two stingless bee species, *M. mondury* and *M. quadrifasciata*, we identified the same five genera as members of the core microbiota: *Lactobacillus* and *Bifidobacterium* were shared with the core microbiota of *A. mellifera*, while the other three genera, *Apilactobacillus*, *Bombella*, and *Floricoccus*, belonged to the core microbiota of only the *Melipona* hosts. Despite these similarities, the two *Melipona* species differed in the prevalence of the core genera; *Apilactobacillus* was more frequent and significantly more abundant in *M. mondury* (Kruskal–Wallis, *P* = 0.0000), whereas *Floricoccus* was more prevalent but not significantly more abundant (*P*=0.7928) in *M. quadrifasciata* as compared to *M. mondury*.

### Differences in non-core members explain variation in community composition within *M. quadrifasciata*

**Fig 5:**
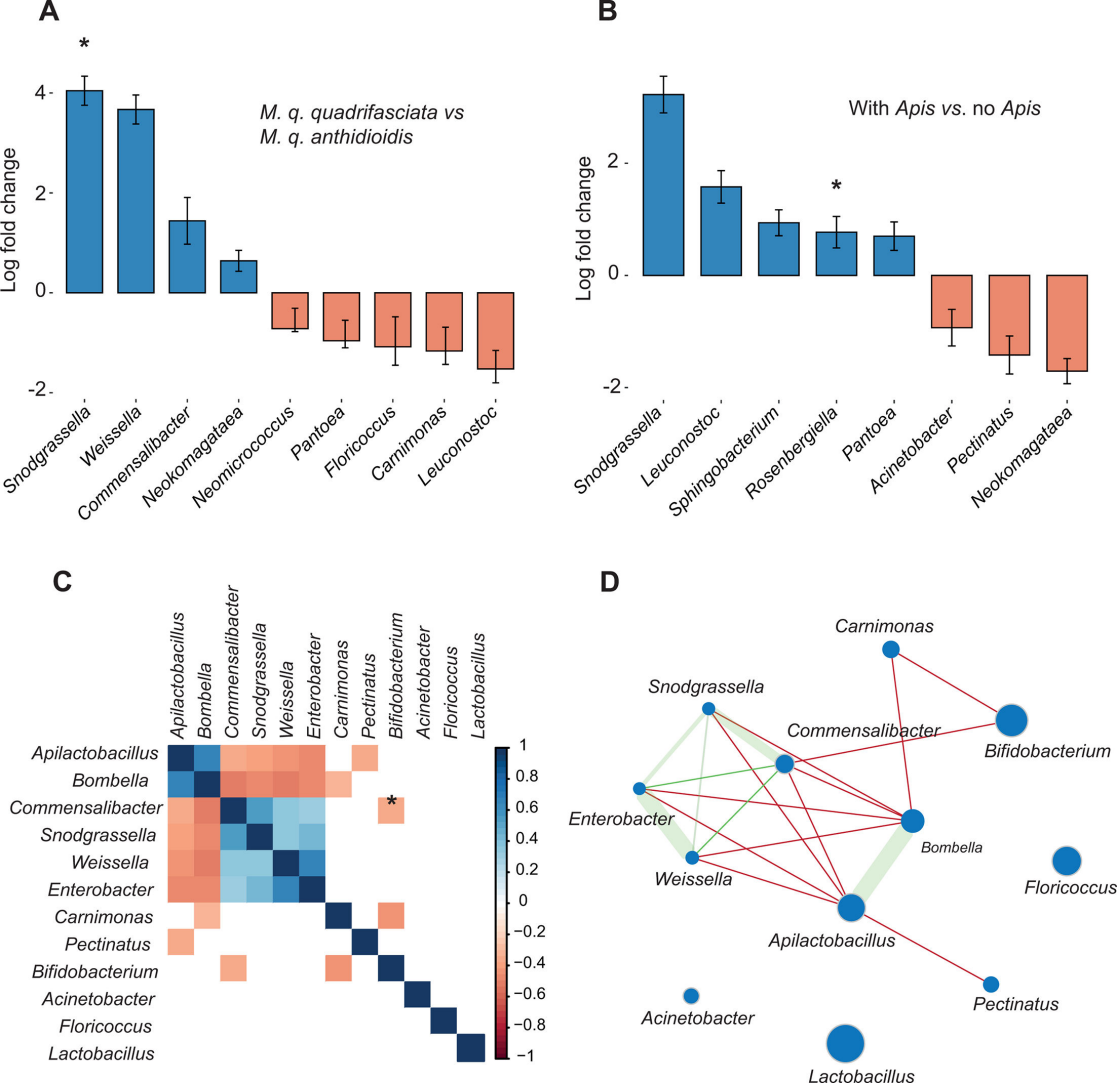
Analyses of bacterial relative abundance in the *M. quadrifasciata* microbiome. (**A**) Differential abundance of bacteria between subspecies. Log fold change indicates the amount of enrichment in the number of reads from a genus in *M. q. quadrifasciata* relative to *M. q. anthidioidis*. Statistically significant log fold changes are indicated by asterisks. (**B**) Differential abundance of bacteria between sites with and without *Apis*. Log fold change indicates the amount of enrichment in the number of reads from a genus in sites where *M. quadrifasciata* is kept with *A. mellifera* relative to sites without *Apis*. (**C**) Matrix of Spearman correlations between relative abundances of bacteria with at least 5,000 reads in the *M. quadrifasciata* data set. (**D**) Network inferred from the statistically significant Spearman correlations. Red and green lines indicate negative and positive correlations, respectively. The width of the green line indicates the intensity of positive correlation.

Although genetic differentiation within *M. quadrifasciata* only marginally explained Bray–Curtis gut community distances (Table 1), we observed clear differences in microbiota composition between the two subspecies of *M. quadrifasciata* in the NMDS analysis. To explore these differences, we performed a differential abundance analysis and found that the gut microbiota of *M. q. quadrifasciata* was enriched in non-core microbiota members, specifically *Weissella* (β = 3.7454, SE = 0.2233, *P* = 0.0000), *Snodgrassella* (β = 3.2946, SE = 0.2526, *P* = 0.0000), and *Commensalibacter* (β = 1.7477, SE = 0.4459, *P* = 0.0021) compared with *M. q. anthidioidis* (Fig. 5A). Because *Snodgrassella* has been reported to be mostly absent from the genus *Melipona* (24), and to test whether habitat could influence its presence via spillover from nearby honey bees, we repeated the analysis comparing colonies kept with versus without *A. mellifera*. Sites where *M. quadrifasciata* co-occurred with honey bees showed enrichment of *Snodgrassella* ASVs, although less pronounced than the subspecies-level contrast (Fig. 5B). Since *A. mellifera* colonies co-occurred with 64% of *M. q. quadrifasciata* but only 37% of *M. q. anthidioidis* samples, part of the apparent effect of *A. mellifera* presence on bacterial abundance may be attributable to host genotype and associated management practices.

To further explore the patterns of relative abundance among non-core taxa in *M. quadrifasciata*, we constructed a co-occurrence network that revealed several significant associations (Spearman’s correlation; *P* < 0.05; Fig. 5C and D). Core bacteria displayed strong positive correlations, particularly between *Apilactobacillus* and *Bombella* (ρ = 0.66), and between *Snodgrassella* and *Commensalibacter* (ρ = 0.54). A positive association was also observed between *Weissella* and *Enterobacter* (ρ = 0.65). In contrast, *Apilactobacillus* and *Bombella* showed consistent negative correlations with *Snodgrassella*, *Commensalibacter*, *Weissella*, and *Enterobacter* (ρ ranging from –0.35 to –0.52), while *Bifidobacterium* correlated negatively with *Commensalibacter* and *Carnimonas* (ρ = –0.35 and –0.44, respectively). Overall, these patterns indicate that core genera tend to co-occur with each other but are negatively associated with non-core genera sporadically present in the gut microbiota of *M. quadrifasciata*.

### Evidence for gut bacteria transfer between the three analyzed bee species

Because all three bee species were sampled from the same geographic regions, we investigated the potential for gut symbiont transfer among them. Although community-level analysis indicated that the overall gut microbiota composition is host specific (Fig. 3A), individual symbionts may occasionally be transferred across species boundaries. The fact that honey bee–specific core members were more prevalent in *M. quadrifasciata* individuals maintained in close proximity to honey bee colonies supports this idea (Fig. 5B).

**Fig 6:**
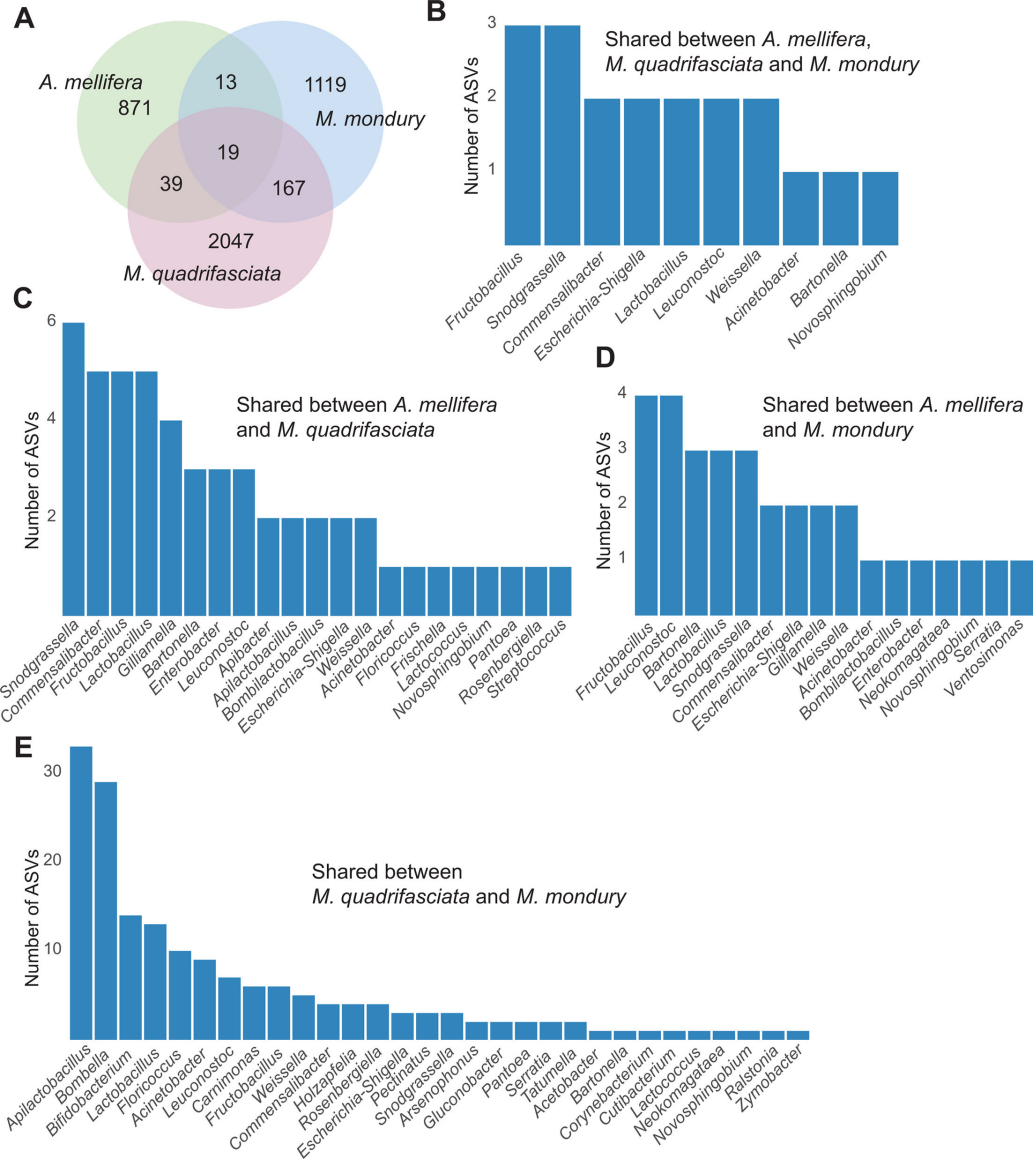
Shared ASVs. (**A**) Venn diagram indicating the number of exclusive and shared ASVs per host species. (**B–E**) Distribution of the number of shared ASVs per bacterial genera.

In line with this, we found that 238 of 4,275 ASVs are shared among the three species: 19 were present across all three, 167 were shared between the two *Melipona* species, and 52 occurred between *A. mellifera* and one *Melipona* species (Fig. 6). The most frequently shared ASVs belonged to *Apilactobacillus* and *Bombella* and were predominantly shared between the *Melipona* species. ASVs shared with *A. mellifera* belonged to various genera, including five of the six honey bee core members (*Lactobacillus*, *Bartonella*, *Bombilactobacillus*, *Gilliamella*, and *Snodgrassella*).

**Fig 7:**
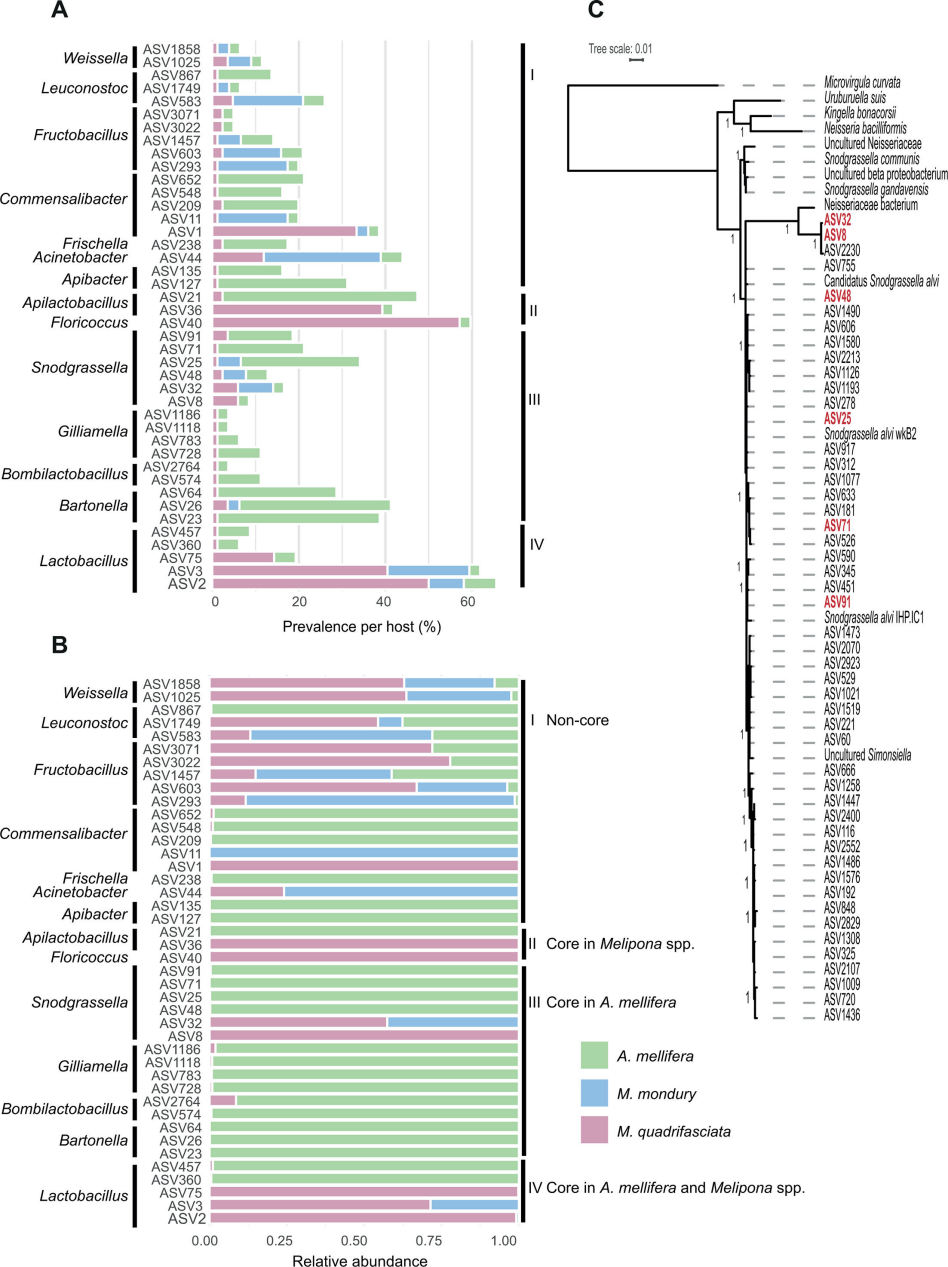
Donor host species of shared ASVs. (**A and B**) Relative prevalence and relative abundance of ASVs shared between *A. mellifera* and *M. quadrifasciata*. For each host species, relative prevalence was calculated as the proportion of samples in which a given ASV was detected, normalized by the total number of samples for that host. Relative abundance was calculated as the proportion of total reads assigned to an ASV in each host, relative to the total number of reads from that ASV across the three host species. (**C**) Phylogeny of *Snodgrassella* ASVs. ASVs shared between *M. quadrifasciata* and *A. mellifera* are shown in red; the triangle highlights a divergent clade containing *Melipona*-specific ASVs.

To investigate how the shared taxa were distributed across the different bee hosts, we focused on 42 genus-assigned ASVs that were detected in both *A. mellifera* and *M. quadrifasciata* (Fig. 7A through C; Table S1). Of these, 19 were also detected in *M. mondury*, allowing us to compare not only between honey bees and stingless bees but also between two closely related stingless bee species. Most ASVs occurred at low prevalence in all hosts, but a subset showed clear and statistically supported host preference. For example, ASV2 and ASV40, assigned to *Apilactobacillus/Lactobacillus*-like lineages, were present in 50% and 57% of *M. quadrifasciata* individuals, respectively, but were rarely detected in *A. mellifera* (≤7.5%; Fisher’s exact test, *P* < 0.05). These ASVs also reached higher relative abundance in *M. quadrifasciata* than in honey bees (Wilcoxon test, *P* < 0.05). ASV36 followed the same pattern; it occurred in ~39% of *M. quadrifasciata* workers but was rarely present in *A. mellifera* samples (2.5%) and was not recovered from *M. mondury* at comparable frequency (Fisher’s exact test, *P* < 0.05). These patterns indicate that some of the shared ASVs are predominantly found in samples of *M. quadrifasciata* and only sporadically occur in honey bees. However, the inverse pattern was also observed. In particular, ASVs assigned to canonical honey bee symbionts such as *Snodgrassella, Gilliamella/Commensalibacter*, and *Bartonella* were enriched in *A. mellifera* as compared to *M. quadrifasciata* samples. For instance, ASV21 was detected in 45% of *A. mellifera* individuals, but only 2.4% of *M. quadrifasciata*; ASV23 in 37.5% vs. 1.2%; ASV127 in 30% vs. 1.2% (Fisher’s exact test, all *P* < 0.05). The same ASVs also reached higher within-host abundance in *A. mellifera* than in *M. quadrifasciata* (Wilcoxon test, *P* < 0.05). Many of these honey bee–biased ASVs were either absent or extremely rare in *M. mondury*, suggesting that these lineages are not broadly established across *Melipona* but instead represent occasional introductions from *A. mellifera*.

Including *M. mondury* in the comparison further showed that not all *Melipona* hosts carry the same set of ASVs. Among the 19 ASVs that were found in *M. mondury*, a few low-prevalence taxa (e.g., *Commensalibacter* ASV11 and *Fructobacillus* ASV293) were detected in ~16% of *M. mondury* samples but in only ~1% of *M. quadrifasciata*, and they reached higher relative abundance in *M. mondury* (Fisher’s exact test and Wilcoxon test, *P* < 0.05). By contrast, high-prevalence *M. quadrifasciata* ASVs such as *Lactobacillus* ASV2 and *Floricoccus* ASV40 were either absent or detected only sporadically in *M. mondury*. This indicates that even between these two closely related *Melipona* species, shared gut bacteria usually have a predominant host species.

### A divergent ASV of *Snodgrasella* shared between stingless bees and honeybees

We examined the shared ASVs of *Snodgrassella* in more detail because this genus is considered a core symbiont of honey bees but has been proposed to be mostly absent in neotropical stingless bees, in particular, the genus *Melipona* (17, 24). Some of the shared *Snodgrassella* ASVs (e.g., ASV25 and ASV71) were significantly more prevalent in *A. mellifera* than in *M. quadrifasciata* (up to 20% vs. ≤3.6% of individuals; Fisher’s exact test, *P* < 0.05) and/or showed higher abundance in *A. mellifera* (ASV25, ASV71, ASV91; Wilcoxon test, *P* < 0.05). They were also nearly identical to the 16S rRNA gene sequence of *Snodgrassella alvi* wkB2 (Fig. 7), the type strain of this *Snodgrassella* species widely distributed in *A. mellifera* (27). Interestingly, two additional *Snodgrassella* ASVs shared between *A. mellifera* and *M. quadrifasciata* (ASV8 and ASV32) were clearly distinct, sharing only 96% sequence identity in the 16S rRNA gene with those of *S. alvi* and forming a divergent deep-branching clade (Fig. 7C). A third ASV (ASV2230) belonging to this divergent clade was detected exclusively in *M. q. quadrifasciata* samples from southern Brazil. Despite their low prevalence and lack of significant enrichment in either stingless bee host relative to the honey bee samples, these ASVs still reached remarkably high relative abundances in three *M. quadrifasciata* samples (41%–77% of all reads) (Fig. S1). Moreover, a *Snodgrassella-*like symbiont previously isolated from the stingless bee species *Frieseomelitta varia* (17) clustered together with ASV8 and ASV32 (Fig. 7C), suggesting that this clade is specific to stingless bees.

## DISCUSSION

Previous studies have shown that stingless bees of the genus *Melipona* harbor specialized gut microbial communities that resemble those of other social bees but remain clearly distinct from the well-characterized microbiota of the honey bee, *A. mellifera* (17, 19, 20, 24, 28, 29). However, the extent to which the gut microbiota of *Melipona* varies across closely related species, subspecies, or individual bees, especially in comparison to that of honey bees, remains unknown. Additionally, it is unclear whether microbial spillover can occur between stingless bee species and honey bees that are kept in close proximity. Here, we addressed these questions by sampling individual adult bees of *M. quadrifasciata*, *M. modury*, and *A. mellifera* from a total of 167 colonies across the geographic range of *M. quadrifasciata* in Brazil. By sampling all three bee species in the same region, we were able to compare their gut microbial diversity while controlling for the environment.

In summary, our results show that both *Melipona* species possess host-specific gut microbial communities that are taxonomically distinct from those of honey bees but exhibit similar levels of within- and between-host diversity. We find evidence for spillover of core microbial members between the three bee species; however, these typically represent minor components of the gut communities.

In contrast to previous studies that characterized the gut microbiota of stingless bees using partial 16S rRNA gene amplicon sequencing of the V3–V4 region, we employed PacBio full-length 16S rRNA gene sequencing. This approach provides higher taxonomic resolution of the resulting ASVs, which enabled us to distinguish closely related bacteria and assess differences in community composition below the bacterial species level within and across the analyzed bee species. As with all DNA-based microbiome surveys, the potential contribution of DNA from non-viable cells cannot be entirely excluded. However, the use of near full-length 16S rRNA gene sequencing reduces this risk, as such longer DNA fragments are unlikely to persist for long extracellularly. In addition, DNA extraction involved enzymatic lysis combined with manual mechanical disruption of gut tissue using a sterile pestle, while avoiding aggressive bead beating to preserve high-molecular-weight DNA required for long-read sequencing. Differences in extraction strategies, including the extent of mechanical disruption, are therefore expected to contribute to variation in community composition across studies.

Our study confirms previous findings that the gut microbiota of social bees is host-specific (15, 17). *M. quadrifasciata*, *M. mondury*, and *A. mellifera* each harbored distinct gut communities Fig. S2). Notably, we detected differences between the two subspecies of *M. quadrifasciata* as well, despite both harboring the same bacterial genera, highlighting the high taxonomic resolution provided by full-length 16S rRNA gene amplicon sequencing. However, our PERMANOVA analyses also revealed that habitat significantly contributes to gut microbiota structure. In fact, habitat explained more of the observed variance than host identity when comparing the microbial communities within *Melipona* samples, particularly between the two subspecies of *M. quadrifasciata*. These results demonstrate that while host specificity remains a major determinant of gut microbiome composition, environmental context is a key source of variation among closely related hosts. However, our analysis provides no evidence that the environment plays a more important role in structuring the gut microbiota of stingless bees, as compared to honey bees. *Melipona* spp. showed neither higher alpha-diversity (Shannon diversity index and Chao1 estimated richness) nor greater beta-diversity dispersion (Bray–Curtis distances) than honeybees across the sampled sites. Only *M. mondury* exhibited slightly higher Chao1 estimates, but the absence of samples from its southernmost distribution and the weak correlation between richness and latitude may have biased comparisons. Previous studies have documented associations between gut bacterial diversity and latitude in both honey bees and stingless bees (30, 31). Our sampling strategy, which relied on single individuals per colony, represents a trade-off between within-colony replication and broader taxonomic and geographic coverage. While smaller per-site sample sizes can increase individual-level variance, the observation of consistent diversity patterns across species and locations suggests that the detected trends are robust. Our primary objective was to test whether Apis species exhibit lower gut microbiota diversity than sympatric stingless bees, rather than to quantify fine-scale within-colony variation.

This broad sampling enabled us to identify the core genera dominating the gut microbiota of the two stingless bee species. We applied an arbitrary cutoff to define core taxa. Therefore, future studies, for example, when conducted across seasons, may come to different results. Our analysis nonetheless offers a quantitative assessment of the most prevalent bacterial groups within the microbiota of the two stingless bee species as compared to honey bees sampled in the same region. The two *Melipona* species shared the same five core genera; however, only two of these (*Lactobacillus* and *Bifidobacterium*) were also part of the core microbiota of honey bees. Although these taxonomic groups are shared across hosts, most ASVs are host-specific, indicating that overlap occurs primarily at higher taxonomic levels rather than through identical strains. Some of the other core genera identified in *Melipona*, such as *Bombella* and *Apilactobacillus*, are also repeatedly detected in honey bees but appear to be less dominant in those hosts. Conversely, Proteobacterial core members typical of honey bees, particularly *Gilliamella* and *Snodgrassella*, are rare in *Melipona* species, although not entirely absent, confirming previous findings (17). Overall, these results indicate that the gut microbiota of *Melipona* is dominated by a distinct set of bacterial core genera compared to honey bees, supporting the idea that the gut microbiota of social bees has undergone substantial compositional changes during evolution.

Despite the host-specific nature of the gut microbiota at the community level in the three bee species, we detected a non-negligible proportion of ASVs (6% of all ASVs) that were present in samples from different host species. The larger fraction of these ASVs was shared between the two *Melipona* species than between *Melipona* and honey bees, suggesting that host phylogeny and ecology may act as barriers to host switching. Although we cannot exclude the possibility that bacteria with the same ASV differ in other parts of their genomes, and hence are evolutionary divergent, these findings suggest that some gut bacteria have been recently transferred between bee species (32, 33). We can also not rule out the chance that some of the detected shared ASVs are artifacts of amplicon sequencing or result from cross-contamination during sample processing. However, the relatively high abundance of several shared ASVs across multiple samples makes such explanations unlikely. To minimize barcode misassignment (including barcode hopping), we applied strict demultiplexing and read-filtering procedures, as detailed in Materials and Methods.

Shared ASVs may represent non-specific, environmental bacteria that are occasionally acquired by different social bee species (e.g*.,* during flower visits), or they could be specialized gut symbionts that are common to multiple host species or primarily associated with one species but occasionally spill over into others. Our enrichment analysis of the 42 genus-assigned ASVs shared between *A. mellifera* and *M. quadrifasciata* showed that many of them belonged to core genera and that they were typically much more prominent in one host versus another. A particularly interesting case was that of *Snodgrassella*. This genus is typically rare in *Melipona* stingless bees (17, 24). However, we detected several *Snodgrassella* ASVs in our *Melipona* samples that were shared with honey bees. Some of these ASVs clustered within a honey bee–specific clade of *Snodgrassella*, and although their abundances were generally low, their presence suggests that this core gut symbiont can spill over from introduced honey bees into native stingless bees. In other cases, however, the shared ASVs belonged to a divergent clade of *Snodgrassella* specific to stingless bees, suggesting that bacterial exchange can occur in both directions. Overall, these results suggest that occasional spillover of core members from their native host into a non-native host does occur. None of the shared ASVs identified here corresponds to canonical opportunistic or pathogenic taxa commonly associated with bee disease. Nevertheless, bacterial taxa that are adapted to one host may have different ecological or physiological effects in a novel host context. In addition, a substantial fraction of ASVs detected in stingless bees remains taxonomically unclassified, precluding functional inference. Our results, therefore, highlight the potential for cross-species bacterial exchange, while underscoring the need for future functional and experimental studies to assess whether such exchanges have consequences for host health.

Although the generally low prevalence and abundance of these ASVs suggest that such bacteria often fail to establish stable associations in the secondary host, likely due to competition with the resident microbiota, our results demonstrate that opportunities for host switching are present. Human beekeeping practices such as mixed apiaries, colony manipulations, or supplemental feeding of stingless bee colonies with honeybee products are likely to increase such opportunities. As with any field-based study, management practices could introduce sources of contamination or transient microbial transfer; however, the consistency of host-specific patterns argues against such artifacts as the primary driver of our results. That stingless bees are susceptible to such environmental factors has been shown in the case of Australian stingless bees, which exhibited changes in gut microbiota composition after their colonies were translocated for pollination services (32, 33). Moreover, recent studies have shown that the gut microbiota of social bees have not strictly co-diversified with their host, but that symbiont gain, loss, and exchanges have occurred (15, 17, 24). This suggests that such ecological opportunities of gut microbiota exchange can result in novel, evolutionary stable associations. Our analyses rely on relative read abundances and do not provide direct estimates of absolute bacterial population sizes. Therefore, future studies combining community profiling with absolute quantification will be required to better assess the biomass and ecological relevance of spillover taxa.

Our study provides a comprehensive strain-level view of the gut microbiota of two important stingless bee species in Brazil and shows that, although *Melipona* communities are compositionally distinct, human-mediated beekeeping creates ecological contact zones that allow gut symbionts to cross species boundaries. We found no evidence that stingless bee microbiota are inherently more environmentally assembled than those of honey bees, but we did detect widespread ASV sharing, including all *A. mellifera* core taxa except *Bifidobacterium,* and discovered divergent *Snodgrassella* lineages thriving in some stingless bee colonies. Our results suggest that management practices such as artificial feeding, colony transport, and mixed apiaries may promote spillover. The ability of such occasionally transferred bacteria to establish themselves in non-native stingless bee hosts and to influence host–microbe or microbe–microbe interactions in the gut warrants further investigation. Whole-genome sequencing and functional studies will be essential to further elucidate the causes and consequences of gut microbiota variation in stingless bees of the genus *Melipona* and specifically to understand how anthropogenic influences reshape bee gut communities and affect pollinator health.

## MATERIALS AND METHODS

### Sampling

All bees were sampled between October 2023 and February 2024 and kept in 100% ethanol at −20°C until use (metadata available in Table S2). Sampling was authorized by SISBIO/ICMBio/MMA through the license #89616-1 to KLH. *M. quadrifasciata* subspecies (*M.q. quadrifasciata* and *M.q. anthidioidis*) were recognized by their differences in abdomen coloration and mitochondrial *cox1* gene haplotypes (34).

### DNA extraction and amplicon sequencing

Abdomens were removed from bee bodies using clean scalpels. The abdomen of one single bee per colony was used for DNA extraction using the Vazyme FastPure Blood/Cell/Tissue/Bacteria DNA Isolation Mini Kit (Nanjing, China). Briefly, individual abdomens were washed twice in 1× PBS, ground with a small sterile plastic pestle in a 1.5 mL tube containing 1× G2 buffer (Qiagen, Hilden, Germany) with 20 mg/mL lysozyme, and then incubated at 37°C for at least 1 h. DNA extraction was performed according to the manufacturer’s protocol. DNA quality and yield were evaluated on a Qubit fluorometer (Thermo Fisher Scientific, Waltham, Massachusetts).

For bacterial amplicon sequencing, the full length of the 16S rRNA gene was amplified from each DNA sample with the KAPA HiFi HotStart polymerase (Roche, Basel, Switzerland), using 1 ng of template DNA, barcoded primers GCATC/barcode/AGRGTTYGATYMTGGCTCAG and GCATC/barcode/RGYTACCTTGTTACGACTT, and following the manufacturer’s instructions. PCR products were pooled, and sequencing libraries were prepared with the SMRTbell Express TPK 2.0 Library Construction system (Pacific Biosciences, Menlo Park, California) using the pooled 16S rRNA gene PCR amplicons. Six negative controls (extraction blanks and PCR no-template controls) were processed and sequenced alongside biological samples. These controls were used to screen for background contamination; control libraries were inspected and subsequently excluded from downstream analyses. No mock community was included in the study. Library preparation, sequencing, data quality control, and demultiplexing were performed at the Lausanne Genome Technologies Facility (UNIL, Lausanne, Switzerland). To minimize barcode misassignment (including barcode hopping), we used sample-specific barcoded primers and applied strict demultiplexing/assignment of reads to samples (performed at the sequencing facility and additionally verified using in-house scripts). Reads with inconsistent or ambiguous barcode assignments were discarded. Barcodes were used on both primers (dual barcoding), which reduces susceptibility to misassignment and allows internal monitoring of unused barcode combinations.

### *M. quadrifasciata* subspecies identification

The main difference between *M. quadrifasciata* subspecies refers to the yellow metasomal stripes, which are continuous in *M. q. quadrifasciata* and discontinuous in *M. q. anthidioides* (35), but this feature is often ambiguous. Therefore, in addition to recording the coloration pattern and the information provided by beekeepers upon sampling, barcoding of the mitochondrial *cox1* gene was used to help classify *M. quadrifasciata* samples in each of the two recognized subspecies. To this end, a DNA aliquot was used to amplify the *cox1* gene with primers BarbeeF (36) and MtD9 (37), generating ~619 bp amplicons. Amplicons were purified with the Exo-CIP Rapid PCR Cleanup Kit (New England Biolabs, Ipswich, Massachusetts) according to the manufacturer’s instructions and then sequenced with the Sanger method by Microsynth (Balgach, Switzerland). For the analysis, the Sanger sequences were assessed for quality and aligned using the Muscle algorithm (38) within Geneious Prime 2025.2.1 (Biomatters, Auckland, New Zealand) and imported into PopART (https://popart.maths.otago.ac.nz/) for haplotype identification. A network was built using the TCS algorithm (39). Subspecies were assigned to separate groups of haplotypes within the network (Fig. 1D) and validated by the information collected at the sampling sites.

### 16S ASV metabarcoding

We used in-house python scripts to assign the demultiplexed sequencing reads to each bee sample. After primer trimming, 16S rRNA gene sequences were filtered for length (between 1,400 and 1,600 bp) and quality (maximum expected error = 2). Amplicon sequence variants (ASVs) were constructed using DADA2 (40) in R version 4.4.0 with default parameters (except errorEstimationFunction = PacBioErrfun and BAND_SIZE = 32). Chimeras were removed from the dereplicated and denoised data set with the removeBimeraDenovo dada2 function, and then, ASVs were assigned to the sequences based on the SILVA database (41) version 138.1 and the dada2 function assignTaxonomy (that uses the RDP Naive Bayesian Classifier algorithm described in Wang et al. [42], with kmer size 8 and 100 bootstrap replicates).

### Phylogenetic analyses

ASVs from the *Melipona* spp. core microbiota were aligned to reference sequences (Table S3) and used for phylogenetic inferences with Geneious Prime 2025.2.1 (Biomatters, Auckland, New Zealand). Alignments were performed using Muscle 5.1 (38), and phylogenies were inferred by maximum likelihood with PhyML (43) using the GTR model with estimated transition/transversion ratios, proportion of invariable sites, and gamma distribution parameters.

### Bacterial community analyses

All statistical analyses were performed in R version 4.4.1 (44) using RStudio version 2024.09.1+394 (Posit Software, Boston, Massachusetts), on a data set from which negative controls and samples with less than 7,000 reads had been removed. Our codes are available at https://github.com/klhaag/metabarcoding-melipona/tree/main/Data_04. To determine whether samples had been sequenced to sufficient depths to capture the true community diversity, rarefaction curves were generated with phyloseq (45) by plotting the number of ASVs observed in each subsample using 7,000 randomizations (“step” = 200). Alpha-diversity estimators (Shannon diversity index and Chao1 estimated richness) were also obtained with phyloseq (45). To test if stingless bees from different species (or subspecies) harbored microbiomes with distinct diversities, we used Kruskal–Wallis tests followed by Dunn tests, and correcting for multiple comparisons with the Benjamini-Hochberg method (FDR). For all statistical analyses, values were considered statistically significant when *P* < 0.05.

Core microbiomes were inferred based on taxa relative abundance and prevalence, as previously suggested (46), defining as core genera present in >70% of individuals and with an average relative abundance of >5%. The top-most abundant and prevalent genera were calculated in order to identify a “common core.” Beta-diversity (Bray–Curtis distance calculated from ASV relative read counts) was used to identify whether the composition of bacterial gut microbiomes differed between species and subspecies. Differences were visualized by non-metric multidimensional scaling (NMDS), and significance was calculated through PERMANOVA using the “adonis2” function from the *vegan* package (47), correcting for multiple comparisons with the FDR method. Both the host species (or subspecies) and the sampling site were used as factors in the analyses, and omega squared (ω^2^) (48) was used to calculate the amount of total variation in microbiota composition explained by each factor (ω^2^ was chosen over r^2^ because it is not sensitive to the number of categories within a factor and can thus be compared across factors). For all PERMANOVA tests, analyses were restricted to municipalities where the species, or haplotypes, under comparison were sampled in sympatry, ensuring that observed differences were not confounded by non-overlapping geographic distributions.

Differential abundance analyses were conducted to identify bacterial genera whose relative representation in *M. quadrifasciata* colonies varied according to (i) mitochondrial haplotype group (sub-species) and (ii) the proximity to *A. mellifera* apiaries. For both comparisons, the filtered 16S rRNA data set was subset to include only *M. quadrifasciata* samples. Statistical analyses were performed at the genus level using ANCOM-BC2 (Analysis of Compositions of Microbiomes with Bias Correction 2) (49), as implemented in the ancombc2 R package. The models were fitted with “Region” or “HapGroup” as fixed effects, applying a prevalence cutoff of 1% (prv_cut = 0.01), FDR correction for multiple testing, and default bias-correction parameters (pseudo_sens = TRUE, s0_perc = 0.05, struc_zero = TRUE, neg_lb = TRUE). Differentially abundant taxa were defined as those showing significant bias-corrected log-fold changes (|LFC|>0, *P*<0.05). For each taxon, ANCOM-BC2 estimates of log-fold change and standard error were extracted and visualized as bar plots relative to the reference group.

In order to incorporate the interactional aspect of the *M. quadrifasciata* core microbiome, focusing on the correlations of the “common core” with less abundant bacterial taxa, a network analysis was carried out with the package NetCoMi (50). Data were preprocessed by separating ASVs associated with each host species and grouping them by their genus. Only bacterial genera with more than 5,000 read counts were considered in the analysis. Networks were made by Spearman correlation, as we do not assume linearity. Correlations were tested for statistical significance by bootstrapping 1,000 permutations and adjusted for multiple tests using FDR adjustment. Only statistically significant relationships were considered (*P* < 0.05).

### Identification of shared taxa and putative symbiont spillover

To identify bacterial lineages shared among host species, we used ASV-level overlap based on the presence/absence matrices of ASVs assigned to each host. The sets of ASVs detected in *A. mellifera*, *M. quadrifasciata*, and *M. mondury* were compared using Venn diagrams. Genera occurring in at least two hosts were defined as shared taxa, while those restricted to one host were considered host-specific. Specifically, 42 genus-assigned ASVs were shared between *A. mellifera* and *M. quadrifasciata*, and among these, 19 were also detected in *M. mondury*. These 42 ASVs were then used for subsequent prevalence and enrichment analyses.

For each ASV and host, prevalence was calculated as the proportion of individual samples positive for that ASV (number of detections divided by total samples per host). Relative abundance was derived from normalized ASV counts per individual, expressed as the fraction of total reads per sample. To prevent zero-inflated distributions and enable median-based comparisons, a small pseudocount (1/12000, approximating one read per library) was added to all abundance values prior to statistical testing. Host bias, defined as the preferential enrichment of an ASV in one host species (the putative donor) relative to another, was evaluated through pairwise differential-abundance comparisons among the three bee species. For each ASV and host pair, we tested prevalence differences using Fisher’s exact tests and abundance differences using Wilcoxon rank-sum tests, with *P*-values adjusted for multiple testing using FDR. An ASV was considered significantly enriched in a host when both prevalence and relative abundance were higher in that host after FDR correction.

## Data Availability

The raw sequences are available at NCBI under BioProject PRJNA1307476.

## SUPPLEMENTAL MATERIAL

Fig. S1 aem.02483-25-s0001.pdf Total number of reads of *Snodgrassella* ASVs.

Fig. S2 aem.02483-25-s0002.pdf Phylogenetic trees of core bacteria.

Table S1 aem.02483-25-s0003.xlsx Prevalence and relative abundance of shared ASVs.

Table S2 aem.02483-25-s0004.xlsx List of bee samples and their associated metadata.

Table S3 aem.02483-25-s0005.xlsx GenBank accession numbers of reference sequences.

ASM does not own the copyrights to Supplemental Material that may be linked to, or accessed through, an article. The authors have granted ASM a non-exclusive, world-wide license to publish the Supplemental Material files. Please contact the corresponding author directly for reuse.
