# Supplementary figures and images for: Conserved gut microbiomes with cross-species spillover between sympatric Neotropical stingless bees and honey bees
Source: Appl Environ Microbiol. 2026 Apr 17;92(5):e02483-25. doi: 10.1128/aem.02483-25 (PMC13188849; doi:10.1128/aem.02483-25)

Figure S1 – Total number of reads of *Snodgrassella* ASVs per sample.

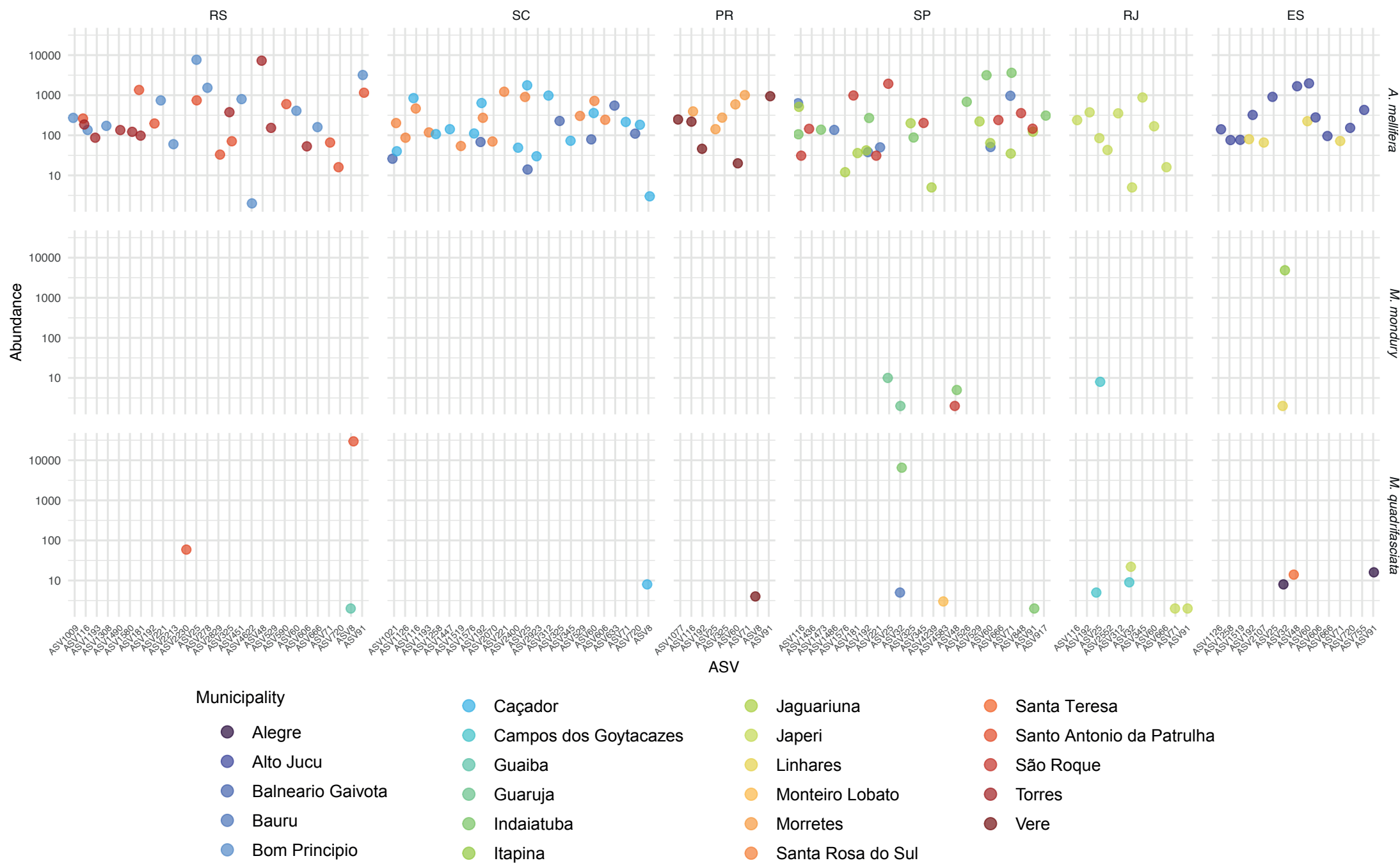

Supplement: Fig. S1 — Total number of reads of Snodgrassella ASVs. [file aem.02483-25-s0001.pdf]
