# Supplementary material for: Conserved gut microbiomes with cross-species spillover between sympatric Neotropical stingless bees and honey bees
Source: Appl Environ Microbiol. 2026 Apr 17;92(5):e02483-25. doi: 10.1128/aem.02483-25 (PMC13188849; doi:10.1128/aem.02483-25)
Supplement: Fig. S2 — Phylogenetic trees of core bacteria. [file aem.02483-25-s0002.pdf]

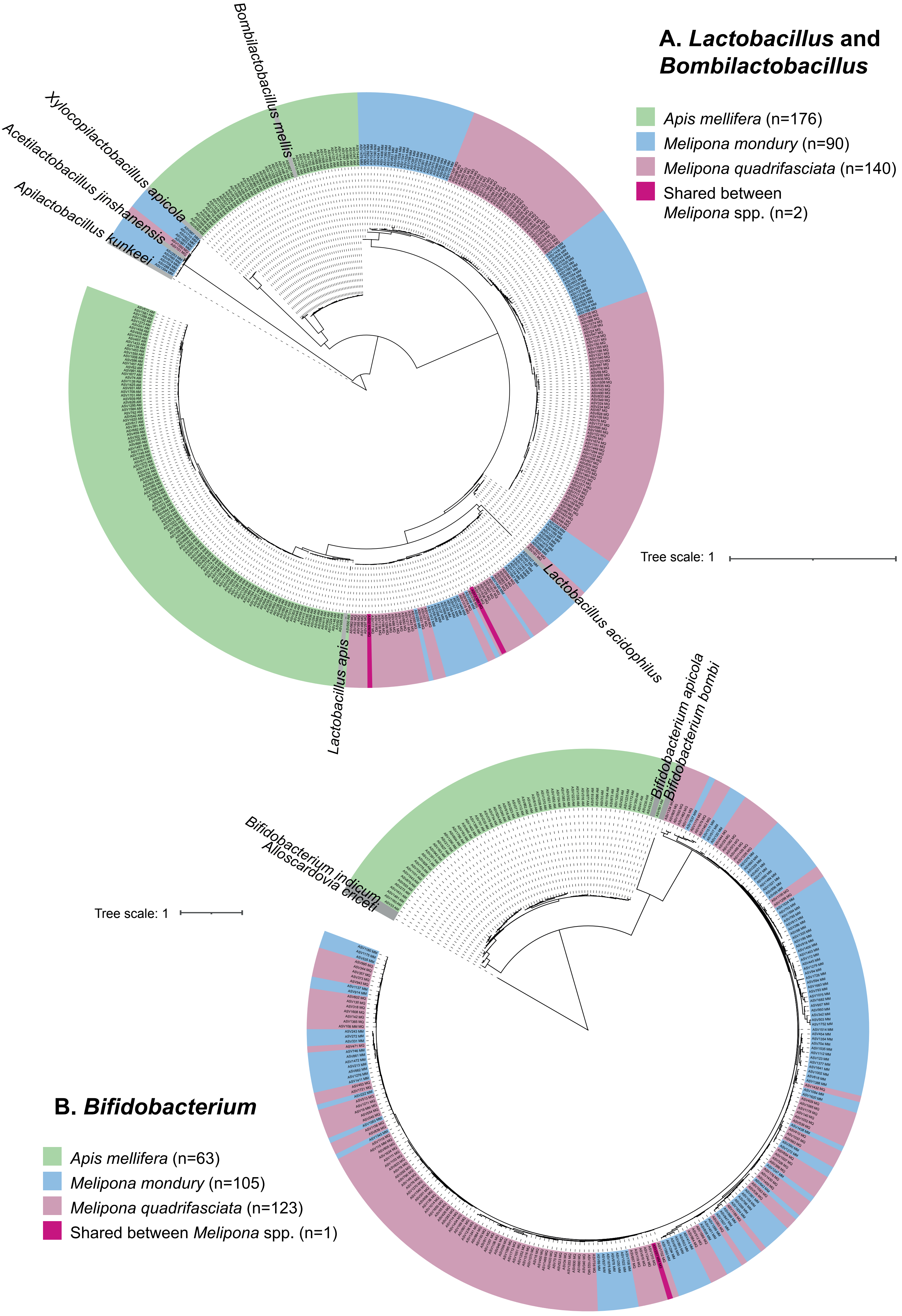
A. Lactobacillus and Bombilactobacillus
Apis mellifera (n=176)
Melipona mondury (n=90)
Melipona quadrifasciata (n=140)
Shared between Melipona spp. (n=2)
Bombilactobacillus mellis
Xylocopilactobacillus apicola
Acetilactobacillus jinshanensis
Apilactobacillus kunkeei
Lactobacillus apis
Lactobacillus acidophilus
Tree scale: 1
B. Bifidobacterium
Apis mellifera (n=63)
Melipona mondury (n=105)
Melipona quadrifasciata (n=123)
Shared between Melipona spp. (n=1)
Bifidobacterium apicola
Bifidobacterium bombi
Bifidobacterium indicum
Alloscardovia criceti
Tree scale: 1

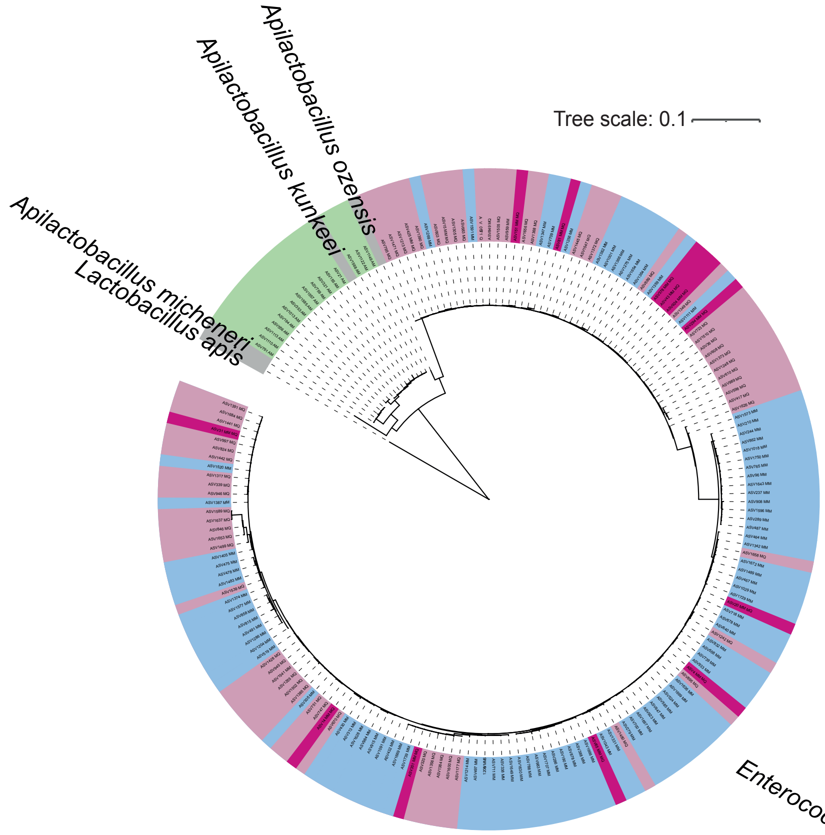

C. *Apilactobacillus*

- *Apis mellifera* (n=16)
- *Melipona mondury* (n=93)
- *Melipona quadrifasciata* (n=60)
- Shared between *Melipona* spp. (n=14)

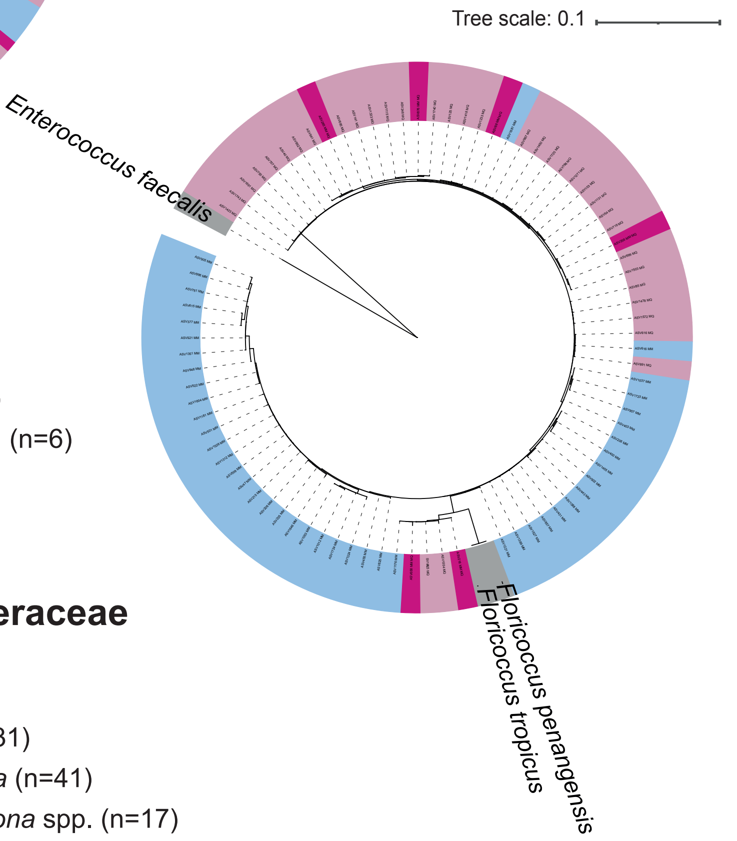

D. *Floricoccus*

- *Melipona mondury* (n=44)
- *Melipona quadrifasciata* (n=35)
- Shared between *Melipona* spp. (n=6)

E. *Bombella* and other Acetobacteraceae

- *Apis mellifera* (n=9)
- *Melipona mondury* (n=31)
- *Melipona quadrifasciata* (n=41)
- Shared between *Melipona* spp. (n=17)

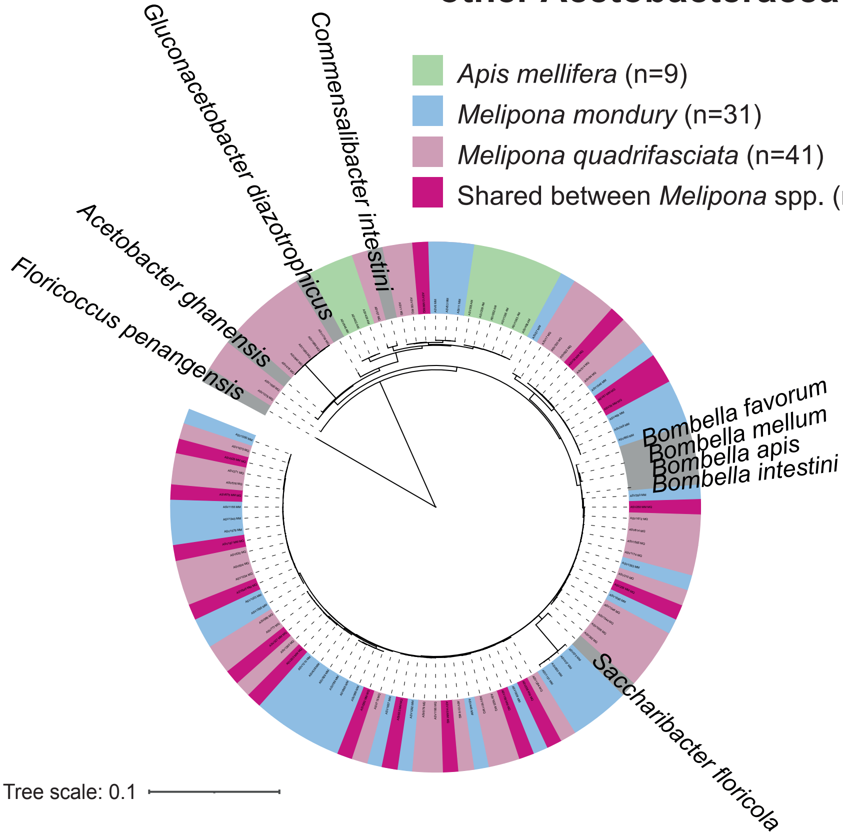

Figure S2 - Phylogenetic trees of core bacteria. Analyses including all *Lactobacillus* and *Bombilactobacillus* (A), *Bifidobacterium* (B), *Apilactobacillus* (C), *Floricoccus* (D), and Acetobacteraceae (E) ASVs with at least 100 reads in samples from each host species.
The number of ASVs with >100 reads identified in each host species is indicated.
